# Correcting for Non-stationarity in BOLD-fMRI Connectivity Analyses

**DOI:** 10.3389/fnins.2021.574979

**Published:** 2021-02-24

**Authors:** Catherine E. Davey, David B. Grayden, Leigh A. Johnston

**Affiliations:** ^1^Department of Biomedical Engineering, University of Melbourne, Melbourne, VIC, Australia; ^2^Melbourne Brain Centre Imaging Unit, University of Melbourne, Melbourne, VIC, Australia

**Keywords:** fMRI, non-stationarity, correlation, connectivity, resting-state, power

## Abstract

In this work fMRI BOLD datasets are shown to contain slice-dependent non-stationarities. A model containing slice-dependent, non-stationary signal power is proposed to address time-varying signal power during BOLD data acquisition. The impact of non-stationary power on functional MRI connectivity is analytically derived, establishing that pairwise connectivity estimates are scaled by a function of the time-varying signal power, with magnitude upper bound by 1, and that the variance of sample correlation is increased, thereby inducing spurious connectivity. Consequently, we make the observation that time-varying power during acquisition of BOLD timeseries has the propensity to diminish connectivity estimates. To ameliorate the impact of non-stationary signal power, a simple correction for slice-dependent non-stationarity is proposed. Our correction is analytically shown to restore both signal stationarity and, subsequently, the integrity of connectivity estimates. Theoretical results are corroborated with empirical evidence demonstrating the utility of our correction. In addition, slice-dependent non-stationary variance is experimentally determined to be optimally characterized by an inverse Gamma distribution. The resulting distribution of a voxel's signal intensity is analytically derived to be a generalized Student's-*t* distribution, providing support for the Gaussianity assumption typically imposed by fMRI connectivity methods.

## 1. Introduction

Functional MRI (fMRI) connectivity analysis utilizes blood oxygen level dependent (BOLD) data to determine how spatially remote brain regions cooperate when a subject is completing a task or is simply at rest. BOLD data is noisy, being impacted by both equipment-related noise, such as coil interference (Bandettini et al., 1998) and scanner drift (Bianciardi et al., 2009), and subject noise, such as physiological interference (Biswal et al., 1995) and subject movement (Barry et al., 2010). Both equipment and subject noise can be irregular and, therefore, non-stationary. Scanners can generate non-stationary signal variance from inhomogeneous radio-frequency (RF) amplification (Tanase et al., 2011), coil resistance (Gudbjartsson and Patz, 1995), disparities in scanner performance over time (Weisskoff, 1996), and signal attenuation from diffusion and dissemination amongst surrounding voxels (Ojemann et al., 1997). *B*_0_ fluctuations exhibit slice dependence due to factors such as respiration effects and cardiac effects (de Moortele et al., 2002), and the slice-dependence of susceptibility-induced signal loss has been recognized and attempts made at compensation (Rick et al., 2010; Anderson et al., 2014). Delay between slice acquisitions can render the introduced non-stationarity slice dependent, which is significant for connectivity in which comparison between voxels is key, in contrast to an activation analysis in which voxels are typically analyzed independently (Kimberg, 2008).

Connectivity analyses in fMRI frequently employ a linear Gaussian model (LGM) to estimate dependence between a seed timeseries and brain voxels (Goebel et al., 2003; Marrelec et al., 2005; Wang and Xia, 2007; Tana et al., 2008). A core assumption of the LGM class is weak stationarity of the constituent timeseries, requiring the mean and variance of the timeseries to be constant over time. Violation of this assumption has implications for the integrity of connectivity results since the distribution of the test statistic is altered (Granger and Newbold, 1974). Linear dependence estimates may also be affected as time-varying variance is analogous to applying a (non-constant) weight to each sample; samples associated with higher variance receive a greater weighting and are consequently more significant in the correlation estimate (Cohen et al., 2003). Random noise occurring at samples accompanied by large variance can alter results and even induce spurious connectivity (Granger and Newbold, 1974). Therefore, if the non-stationarity remains uncorrected, connectivity results may not be reflective of the underlying linear dependence between voxels.

Non-stationarity of BOLD timeseries has been modeled in an effort to resolve the loss of integrity associated with a stationarity assumption imposed on non-stationary data. Lund et al. (2006) modeled non-stationarity of noise via nuisance variable regression in an fMRI activation analysis. They incorporated cosine regressors for low-frequency drift resulting from hardware instabilities, first order Volterra expansion regressors to model rigid subject movement, and harmonic regressors to model aliased physiological noise. This approach restricts removal of non-stationary effects to noise processes explicitly modeled in the regressors. Diedrichsen and Shadmehr (2005) addressed time-varying volume variance in a fMRI activation study by employing a linear model with Gaussian noise characterized by time dependent variance. The non-stationary variance process was empirically determined to apply globally, so that sample variance was estimated for each image volume, and employed as a weight in a least squares estimate of regression coefficients. Luo and Puthusserypady (2007) consider the noise model proposed by Diedrichsen and Shadmehr (2005), addressing robustness concerns regarding estimation of the noise covariance matrix by employing a Bayesian framework to estimate the time-varying weights. Oikonomou et al. (2010) introduced a model of non-stationary noise for BOLD activation analyses that assumed that image slices contained unique stationary variance, applied as weights to the non-stationary noise variance of each voxel; the spatial extent of the non-stationary noise was not explored.

The necessity for modeling non-stationarity of BOLD timeseries has often been argued with visual evidence (Calhoun et al., 1999; Diedrichsen and Shadmehr, 2005; Lund et al., 2006) or otherwise assumed (Long et al., 2005; Oikonomou et al., 2009, 2010), so that there has been a notable absence of statistical evidence demonstrating non-stationarity (Park et al., 2010). Turner and Twieg (2005) established the temporal non-stationarity of independent component analysis (ICA) components derived from fMRI noise and found evidence for the persistence of the spatial components, although what these components represented is not stated. Furthermore, modeling of non-stationary processes has historically been limited to fMRI activation data; until recently there had been few investigations into non-stationarity in resting state fMRI and existent studies focussed on addressing the non-stationarity of noise processes (Calhoun et al., 1999; Diedrichsen and Shadmehr, 2005; Lund et al., 2006).

More recently, there has been increased interest in examining the non-stationarity of functional connectivity in resting state fMRI, such as Hindriks et al. (2016), Long et al. (2005), Chang and Glover (2010), Niazy et al. (2011), and Park et al. (2010), with a corresponding attempt to discern if the evident time variance is of neural or physiological origin. Handwerker et al. (2012) examined the non-stationarity of brain correlations, demonstrating the existence of periodic fluctuations in correlation at distinct frequencies, noting that the origin of the fluctuations remains unclear, which limits the basis for neural interpretation. Lindquist et al. (2014) examine the use of sliding window correlation as a means to reveal non-stationarity in underlying BOLD connectivity. Cribben et al. (2014) present a method for identifying distinct periods with respect to changing connectivity patterns. Allen et al. (2012) employ both sliding window correlation and spatial ICA to identify time-varying correlation, and a k-means clustering technique to isolate reoccurring connectivity patterns, though spatial stationarity is assumed. Hutchison et al. (2013) review recent the literature discussing non-stationarity of functional connectivity in resting-state fMRI, specifically examining sliding-window correlation and the non-stationarity of spatial ICA components. Despite the recent interest in time-varying connectivity, there has been an absence of discussion regarding the potential impacts of time-varying signal power.

In this paper, we correct fMRI connectivity estimates for non-stationarity induced by time-varying signal power during BOLD data acquisition. Experimental fMRI data is empirically shown to contain time-varying slice variance that is optimally characterized by an inverse gamma distribution. The resulting distribution of voxel intensity values is analytically determined to be a generalization of Student's-*t* distribution. On the basis of these empirical results, a model of slice-dependent non-stationarity is introduced, and an analytic derivation of sample correlation between non-stationary timeseries is presented. It should be noted that the proposed model captures non-stationarity induced by slice-dependent, time-varying signal power, and is not intended to be a universal model of all sources of non-stationarity in the data. We demonstrate the capacity for time-varying weights to alter both the magnitude and, in specific cases, the sign of correlation estimates. A straightforward correction to voxel timeseries affected by time-varying signal power is proposed, and is analytically shown to both restore signal stationarity, and to ameliorate the impact of non-stationary signal power on subsequent linear dependence estimates. Theoretical results are corroborated with empirical evidence that demonstrates the utility of our correction in restoring stationarity and the integrity of connectivity results.

This paper is organized as follows. Theoretical results are detailed in section 3.1, incorporating a description of the non-stationary signal model, with a derivation for the distribution of voxel intensity for signals with time-varying variance (subsection 3.1.1), and the consequent expression for correlation between non-stationary timeseries with proposed correction (subsection 3.1.2). Empirical and experimental methods are described in section 2, followed by a description of results in section 3 and discussion in section 4.

## 2. Methods

We examine the impact of non-stationary signal power multiplicatively applied to underlying stationary signals during acquisition. In order to avoid confusion, we introduce a nomenclature to describe the multiple sources of signal variance. Underlying stationary timeseries are associated with a constant variance, which will be referred to as stationary signal variance. Time-varying signal power, modeled as a slice-dependent, non-stationary variance, will be referred to as slice dependent non-stationary variance or, equivalently, as time-varying signal power. Sample variance of voxel intensities within a slice will be referred to as sample slice variance and, similarly, sample variance of voxels within a specific tissue type will be referred to as sample tissue variance. Finally, variance of sample correlation will be explicitly identified as such.

### 2.1. Simulated Data

MATLAB was used to generate simulation datasets of 2, 000 timeseries pairs. Denote the constituent timeseries in each pair by $x_{m,t}^{\left(st\right)}$ and $y_{n,t}^{\left(st\right)}$, where *m* and *n* index slice number. Each pair was generated from a bivariate normal distribution characterized by: timeseries length, *T* = 500; underlying correlation, ρ = 0.3; mean values of $x_{m,t}^{\left(st\right)}$ and $y_{n,t}^{\left(st\right)}$, denoted μ_*x*_ and μ_*y*_, respectively, and drawn from the range [−100, 100]; and stationary signal variance of $x_{m,t}^{\left(st\right)}$ and $y_{n,t}^{\left(st\right)}$, denoted σ_*x*_ and σ_*y*_, respectively, both randomly selected from the range (0, 10]. For each dataset, a slice dependent non-stationary variance was randomly generated for timeseries in slice *m*, i.e., $x_{m,t}^{\left(st\right)}$, and another for timeseries in slice *n*, $y_{n,t}^{\left(st\right)}$, to emulate the time-varying signal power associated with acquisition of voxels within a slice, and denoted $\sigma_{m,t}^{2}$ and $\sigma_{n,t}^{2}$, respectively. Samples for each time point of the time-varying power processes were randomly drawn from an inverse gamma distribution, which was characterized by two parameters randomly drawn from the range [1, 10]. Each timeseries in the dataset slice was then weighted by the square root of the appropriate time-varying signal power according to whether it was designated $x_{m,t}^{\left(st\right)}$ or $y_{n,t}^{\left(st\right)}$, within the pair, to generate the non-stationary signals $x_{m,t}$, and $y_{n,t}$, respectively.

Slice dependent non-stationary variance was estimated for each slice by calculating sample variance spatially, across the 2, 000 $x_{m,t}^{\left(st\right)}$ signals, and separately for the 2, 000 $y_{n,t}^{\left(st\right)}$ signals, to emulate the number of voxels that would typically be available in a fMRI dataset. The precision correction, Equation (12), was applied to generate timeseries corrected for non-stationarity of the form proposed in Equation (2). Correlation was calculated between each timeseries pair before being weighted by time-varying signal power, after being weighted, and after the precision correction was applied.

In order to determine the impact of both time-varying signal power, and our subsequent correction, on autocorrelated signals, we followed the same technique as for the white signals above, but generated the signals using a vector autoregression (VAR) model. In this case,

(1) $$\begin{array}{l} \left[\begin{array}{c} x_{m,t}^{\left(st\right)}\\ y_{n,t}^{\left(st\right)} \end{array}\right]=\left[\begin{array}{c} v_{x}\\ y_{x} \end{array}\right]+{\text{A}}_{1}\left[\begin{array}{c} x_{m,t-1}^{\left(st\right)}\\ y_{n,t-1}^{\left(st\right)} \end{array}\right]\;+. . .+{\text{A}}_{\text{p}}\left[\begin{array}{c} x_{m,t-p}^{\left(st\right)}\\ y_{n,t-p}^{\left(st\right)} \end{array}\right]\;+\epsilon ,\\ \;\epsilon \;∼\;N\;\left(0,\;\left[\begin{array}{cc} \sigma_{x}^{2} & \rho \sigma_{x}\sigma_{y}\\ \rho \sigma_{x}\sigma_{y} & \sigma_{x}^{2} \end{array}\right],\right) \end{array}$$

where ν_*x*_ and ν_*y*_ allow for timeseries with non-zero mean, **A_p_** are the 2 × 2 matrices of coefficients at the designated lag, *p*, $x_{m,t-p}^{\left(st\right)}$ is the value of the stationary timeseries at lag *p* and **ϵ** is a noise vector with Gaussian distribution as specified.

We generated random VAR models using randomly selected model order, *p* ∈ [1, 3], and all other values the same as for the white signals above, including $\sigma_{x}^{2}$ and $\sigma_{y}^{2}$ randomly drawn from the range (0, 10], and both ν_*x*_ and ν_*y*_ randomly set so that μ_*x*_ and μ_*y*_ are in the range [−100, 100], where $\mu ={\left(I-A_{1}-\cdots -A_{p}\right)}^{-1}\left[\begin{array}{c} \nu_{x}\\ \nu_{y} \end{array}\right]$, for $\mu ={\left[\mu_{x},\mu_{y}\right]}^{\prime }$. The **A**_*p*_ matrices were specified via random generation of the poles of each VAR(*p*) model with magnitude <1, and each model tested for stability.

We followed the same precision correction as for the white signals, in weighting the timeseries with time-varying variance according to whether the signal was allocated to slice *m* or *n*, and applying the precision correction to generate timeseries corrected for non-stationarity in signal power.

### 2.2. Experimental Data

Two distinct datasets were collected. For the first dataset, two healthy subjects were scanned on a 3T Siemens Tim TRIO MRI scanner using two different procedures: (1) resting state BOLD echo planar imaging, and (2) a motor task performance BOLD EPI. For both procedures, we collected 219 volumes for each participant (repetition time = 1, 600 ms; echo time = 20 ms; flip angle = 90°; 24 trans-axial slices, each 5.5mm thick, matrix = 64 × 64, field of view [FOV] = 200 × 200 mm^2^; acquisition voxel size=3.125 × 3.125 × 5.5 mm^3^). The motor task was a block design containing alternating periods of 30s each. During the active period, the subjects were instructed to press either the left or right button, according to an arrow displayed on the screen that changed randomly every second. During the baseline period, subjects were instructed to focus on a crosshair at the screen's center point. This dataset will be referred to as dataset 1.

For the second dataset three additional subjects were scanned on a 3T Siemens Tim TRIO MRI scanner using resting state BOLD echo planar imaging. We collected 240 volumes for each subject (repetition time = 2, 000 ms; echo time = 20 ms; flip angle = 90°; 91 trans-axial slices, each 5 mm thick, matrix = 91 × 109, field of view [FOV] = 200 × 200 mm^2^; acquisition voxel size=3.125 × 3.125 × 5.5 mm^3^). This dataset will be referred to as dataset 2.

All images were motion corrected and smoothed using a 2D isotropic Gaussian 6mm kernel, applied using MATLAB. A conventional general linear model (GLM) activation analysis (Friston et al., 1994) was applied to the motor task data; the maximum t-statistic in the right hemisphere primary motor cortex (RMC) region was used to identify the RMC. An average hemodynamic timeseries was created for the RMC of each subject by averaging across voxels in the region of interest (ROI).

For each subject, slice dependent non-stationary variance was estimated by calculating the sample slice variance of voxel intensities in each slice at each time point. The best-fit distribution for the sample slice variance of each slice was determined by calculating the maximum likelihood estimates (MLEs) for distribution parameters and identifying the distribution that attained the minimum negative log-likelihood amongst the set of Weibull, Gaussian, Gamma, Inverse Gamma, Student's-t, Exponential, Log-normal, Laplace, and Rayleigh distributions. The Wilcoxon signed-rank test was used to determine if sample slice variances of different slices were drawn from distributions with equal mean (Wilcoxon, 1945). This choice was motivated by the non-normal distribution of slice variance. The test was applied pairwise between all slices. The null hypothesis was that the median difference when comparing sample slice variances between two slices, is zero, while the alternative hypothesis was that the median difference is non zero. If the null hypothesis was rejected, the samples for the two slice variances being examined could be considered to be drawn from different distributions. Sample slice variance was also tested for stationarity, for which the augmented Dickey Fuller test was employed (Said and Dickey, 1984). In both cases, the results were significance tested with α = 0.01.

To establish whether tissue type is a significant determinant of slice dependent variance, tissue masks were generated for each subject using the FSL software suite. For each image volume, the sample tissue variance across all voxels associated with a specific tissue type was calculated, yielding a timeseries of volume dependent variance for each tissue, including gray matter, white matter, and cerebrospinal fluid (CSF). Given that sample tissue variance and sample slice variance were necessarily generated from a common set of voxels, to establish whether tissue type is a significant determinant of slice-dependent variance, whole-volume variance was calculated at each time point and regressed out from both the tissue variance timeseries and the slice variance timeseries. The resulting tissue variance timeseries were correlated with slice variance timeseries, and tested for significance using a Student's *t*-test. Additionally, to establish whether the ratios of tissue types within each slice impacted the sample slice variance, the optimal inverse gamma parameters for each slice variance process were correlated with the proportion of each specific tissue type in each slice, and tested for significant using a Fisher's-*z* transform.

Corrected voxel timeseries were formed by weighting voxel samples using the slice dependent variance, as an estimate of time-varying signal power. Seed-voxel correlation maps were generated with a RMC seed for datasets with and without applying the correction for time-varying signal power. Correlation estimates were significance tested using Fisher's z-transformation (Fisher, 1915), with degrees of freedom corrected for dependent samples (Davey et al., 2013), and the resulting maps thresholded (α = 0.01, Bonferroni corrected).

## 3. Results

### 3.1. Theoretical Results

In the acquisition of EPI BOLD data, two-dimensional slices are usually obtained serially, on a millisecond time scale. This can result in changes in the equipment and subject environment, ultimately modifying signal characteristics between slices, resulting in non-stationarity of voxel timeseries (Calhoun et al., 1999). The slice dependence of the non-stationary processes impacting fMRI data is verified experimentally in section 2.2. Voxels within the same slice are acquired simultaneously and hence signal power within a slice is constant.

fMRI analyses typically assume stationarity of voxel timeseries and therefore it is desirable to determine a correction to restore stationarity. We propose a model of non-stationarity for resting state BOLD data in which each two-dimensional slice variance is characterized by a distinct time-varying signal power. The distribution of voxel intensity is analytically derived for this model and the impact of the non-stationarity on correlation is determined. Crucially, a simple correction is proposed that is analytically shown to restore stationarity and correct correlation values so that they reflect the underlying linear dependence between voxel timeseries.

#### 3.1.1. A Non-stationary Model of Resting State Timeseries

We begin by proposing a model for the measured, non-stationary signal in a voxel *x* situated in slice *n*. The form of the model was motivated by the need to account for slice-dependent signal power in conjunction with the experimental results detailed in section 3. The signal model decomposes the voxel intensity into stationary and non-stationary components,

(2) $$\begin{array}{r} x_{m,t}=x_{m,t}^{\left(st\right)}\sigma_{m,t},\;t=1,\ldots ,T, \end{array}$$

where $x_{m,t}^{\left(st\right)}$ denotes the stationary signal component at time *t*, and σ_*m, t*_ is a time-varying multiplicative variance process associated with slice *m*, and distributed across time according to an inverse gamma distribution,

(3) $$\begin{array}{r} \sigma_{m,t}^{2}\sim IG\left(\alpha_{m},\frac{1}{\beta_{m}}\right). \end{array}$$

Here α_*m*_ and β_*m*_ are slice specific shape and scale parameters, respectively. Consequently, slice dependent non-stationary variance has a mean of $\frac{1}{\beta_{m}\left(\alpha_{m}-1\right)}$, for β_*m*_ > 0, α_*m*_ > 2.

We now determine the distribution of non-stationary voxel intensity, $x_{m,t}$, when σ_*m, t*_ is unknown (Equation 2). The resting state stationary component, $x_{m,t}^{\left(st\right)}$, is assumed to be Gaussian distributed (Wink and Roerdink, 2006),

(4) $$\begin{array}{r} x_{m,t}^{\left(st\right)}\sim N\left(0,\sigma_{x}^{2}\right). \end{array}$$

As $x_{m,t}^{\left(st\right)}$ and σ_*m, t*_ are independent random variables, at each time point a voxel in slice *m* has distribution

(5) $$\begin{array}{r} x_{m,t}\sim N\left(0,\sigma_{x}^{2}\sigma_{m,t}^{2}\right). \end{array}$$

The distribution of voxel intensity as derived in Appendix A is given by

(6) $$\begin{array}{r} p\left(x_{m,t}\right)=\frac{\Gamma \left(\alpha +\frac{1}{2}\right)}{\Gamma \left(\alpha \right)}{\left(\frac{1}{2\pi \beta \sigma_{x}^{2}}\right)}^{\frac{1}{2}}{\left(1+\frac{{x_{m,t}}^{2}}{2\sigma_{x}^{2}\beta }\right)}^{-\left(\alpha +\frac{1}{2}\right)}. \end{array}$$

The voxel intensity distribution (Equation 6), is an instance of the generalized Student's-*t* distribution (Härdle and Simar, 2007, p.129) with parameterization μ_*t*_ = 0, ν_*t*_ = 2α, and $\sigma_{t}^{2}=\frac{\beta }{\alpha }\sigma_{x}^{2}$, where subscript *t* denotes a Student's-*t* distribution parameter. Consequently, the voxel intensity of sampled non-stationary resting state BOLD data described by the proposed model in Equations (2)–(3) is characterized by a generalized Student's-*t* distribution.

#### 3.1.2. Impact of Non-stationary Signal Power on fMRI Connectivity

Correlation-based measures assume stationarity of timeseries and are known to be susceptible to spurious significance if the stationarity assumption is violated (Granger and Newbold, 1974). We derive expressions for both correlation between non-stationary timeseries to determine the impact of time-varying noise on estimates of connectivity. In Appendix B sample correlation was derived as

(7) $$\begin{array}{r} \text{corr}\left(x_{m,t},y_{n,t}\right)=\kappa \text{ corr}\left(x_{m,t}^{\left(st\right)},y_{n,t}^{\left(st\right)}\right), \end{array}$$

where

(8) $$\begin{array}{r} \kappa =\frac{\mu_{\sigma_{m,t}\sigma_{n,t}}}{\sqrt{\mu_{\sigma_{m,t}^{2}}\mu_{\sigma_{n,t}^{2}}}}, \end{array}$$

and by the Cauchy-Schwarz inequality (Cauchy, 1921),

(9) $$\begin{array}{r} |\kappa |\leq 1. \end{array}$$

Consequently, time-varying signal weights necessarily reduce the magnitude of correlation between non-stationary signals, where the extent of the reduction depends on the similarity of the time-varying weight processes. It is important to note that it is feasible for the time-varying processes to modify the sign of the correlation in the event that the weights are drawn from the set of real numbers. In such a case, correlation may appear as anti-correlation and vice-versa.

The variance of sample correlation was found in Appendix B to be

(10) $$\begin{array}{r} var\left(\text{corr}\left(x_{m,t},y_{n,t}\right)\right)\approx \frac{1-\kappa^{2}\text{corr}{\left(x_{m,t}^{\left(st\right)},y_{n,t}^{\left(st\right)}\right)}^{2}}{T-2}. \end{array}$$

Since κ ≤ 1, the variance of sample correlation between non-stationary timeseries will be larger than that of stationary signals. Consequently, estimates of correlation will be more prone to spurious significance.

#### 3.1.3. Correcting fMRI Connectivity for Non-stationary Signal Power

Several fMRI connectivity methods, such as correlation-based measures (Friston et al., 1994; Wang and Xia, 2007), Granger causality methods (Goebel et al., 2003; Chen et al., 2006; Guo et al., 2008), and structural equation modeling (McIntosh and Gonzalez-Lima, 1994), assume stationarity of voxel timeseries. If BOLD data is acquired with non-stationary signal power, this assumption is violated. We now propose a correction to apply to voxel timeseries generated from time-varying signal power of the form proposed in our model (Equation 2). The correction is analytically shown to restore stationarity in the event of such time-varying power during image acquisition.

Let $x_{m,t}^{\left(c\right)}$ denote a corrected voxel timeseries given by

(11) $$\begin{array}{r} x_{m,t}^{\left(c\right)}=\frac{x_{m,t}}{{\hat{\sigma }}_{n,t}}, \end{array}$$

where ${\hat{\sigma }}_{n,t}^{2}$ is the sample slice variance of voxel intensities within slice *m* at time *t*. It is assumed that this sample slice variance will include both the time-varying signal power of interest, $\sigma_{m,t}^{2}$, and a stationary signal variance component, or scale factor, σ_*m*_, such that

(12) $$\begin{array}{l} x_{m,t}^{\left(c\right)}=\frac{x_{m,t}^{\left(st\right)}\sigma_{m,t}}{\sigma_{m,t}\sigma_{m}}\\ \;=\frac{x_{m,t}^{\left(st\right)}}{\sigma_{m}}. \end{array}$$

Observe that the corrected voxel timeseries is now stationary, but with a variance that differs by a constant factor from that of the underlying stationary signal variance component, $x_{m,t}^{\left(st\right)}$ (Equation 4). Consequently,

(13) $$\begin{array}{r} x_{m,t}^{\left(c\right)}\sim N\left(0,\frac{\sigma_{x}^{2}}{\sigma_{m}^{2}}\right). \end{array}$$

Correlation between corrected timeseries is given by

(14) $$\begin{array}{l} \begin{array}{c} \text{corr}\left(x_{m,t}^{\left(c\right)},y_{n,t}^{\left(c\right)}\right)=\frac{1}{T}\overset{T}{\underset{t=1}{\sum }}\left(\frac{x_{m,t}^{\left(st\right)}}{\frac{\sigma_{x}}{\sigma_{m}}}\right)\left(\frac{y_{n,t}^{\left(st\right)}}{\frac{\sigma_{y}}{\sigma_{n}}}\right)\\ \;=\text{corr}\left(x_{m}^{\left(st\right)},y_{n}^{\left(st\right)}\right), \end{array} \end{array}$$

where the first line is a direct result of the definition of corrected timeseries in Equation (12). This demonstrates that correlation between timeseries with time-varying signal power, corrected for this source of non-stationarity, is equivalent to correlation between the underlying stationary voxel timeseries. The impact of the non-stationary signal weighting has been removed and the intrinsic linear dependence has been recovered, as required.

### 3.2. Simulation Results

**Figure 1:**
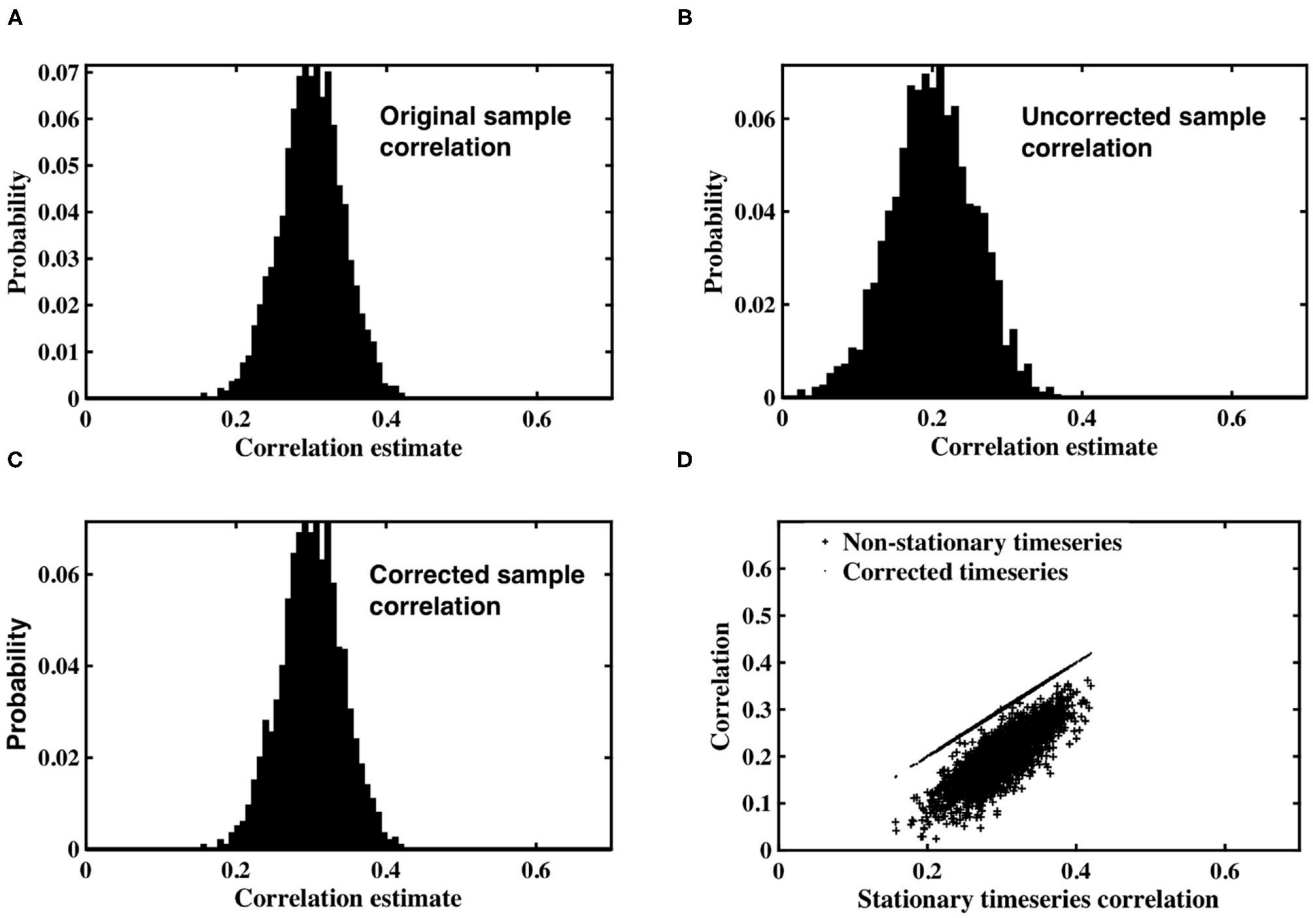
**(A)** Histogram of correlation estimates between stationary timeseries pairs with underlying correlation, ρ = 0.3. **(B)** Histogram of correlation estimates acquired from non-stationary timeseries pairs, with time-varying signal power for each timeseries within a pair randomly derived from *IG*(2, 2) or *IG*(3, 2). **(C)** Histogram of correlation between timeseries pairs corrected for non-stationarity. **(D)** Scatter plots between original correlation estimates drawn from stationary timeseries against correlation estimates of weighted, non-stationary timeseries (crosses) and correlation estimates derived from timeseries corrected to restore stationarity (dots).

Simulated datasets were used to examine the impact of spatially dependent non-stationary variance on sample correlation, as well as the utility of the timeseries correction proposed in Equation (12). Pairs of correlated, stationary timeseries were generated using the methods described in section 2.1, and sample correlation calculated between each pair. A histogram of the correlation estimates is shown in Figure 1A, in which the expected value of sample correlation matches the underlying true correlation value of 0.3. Each stationary timeseries in a pair was weighted by one of two time-varying inverse Gamma variance processes, simulating time-varying signal power, depending on whether the timeseries was designated as coming from slice *m*, or slice *n*. A histogram of correlation estimates acquired for the non-stationary pairs is shown in Figure 1B. The expected value of sample correlation between the non-stationary timeseries is clearly diminished, having a value of 0.48, in precise agreement with the analytic expression derived in Equation (7). Furthermore, the variance of the correlation estimates for the weighted, non-stationary timeseries is increased, thereby reducing confidence in the estimates. Consequently, if left uncorrected, the non-stationarity has the propensity to induce spurious correlation.

The non-stationary timeseries were corrected by an estimate of slice variance at each time point, according to the simple correction proposed in Equation (12). Sample correlation was calculated between corrected timeseries pairs (Figure 1C). The results clearly demonstrate the efficacy of our correction in restoring stationarity to the timeseries, and re-establishing the expected value of correlation estimates to the true, underlying correlation of 0.3.

Figure 1D contains a scatterplot between sample correlation values calculated between the stationary timeseries, against both sample correlation estimates between the non-stationary timeseries, and those between the corrected timeseries. While the histograms are indicative of changes in the distribution of sample correlation, the scatterplots depict changes in individual correlation estimates as a consequence of the time-varying weighting and subsequent correction. Correlation estimates of weighted timeseries differ by up to 0.25 from the correlation contained in the original, stationary timeseries. Conversely, the scatterplot between correlation estimates from the stationary timeseries, and those derived from timeseries corrected by estimates of the time-varying weights, are almost indistinguishable.

The impact of the autocorrelation of fMRI timeseries on our proposed correction was examined. Pairs of stationary VAR timeseries were generated according to Equation (1), and one timeseries within each pair allocated to slice *m* or *n*. Each VAR timeseries within a slice was weighted with a time-varying signal power, generated according to an inverse gamma distribution. The non-stationary variance was estimated for each slice, the VAR timeseries precision corrected, and sample correlation recalculated for each VAR timeseries pair.

**Figure 2:**
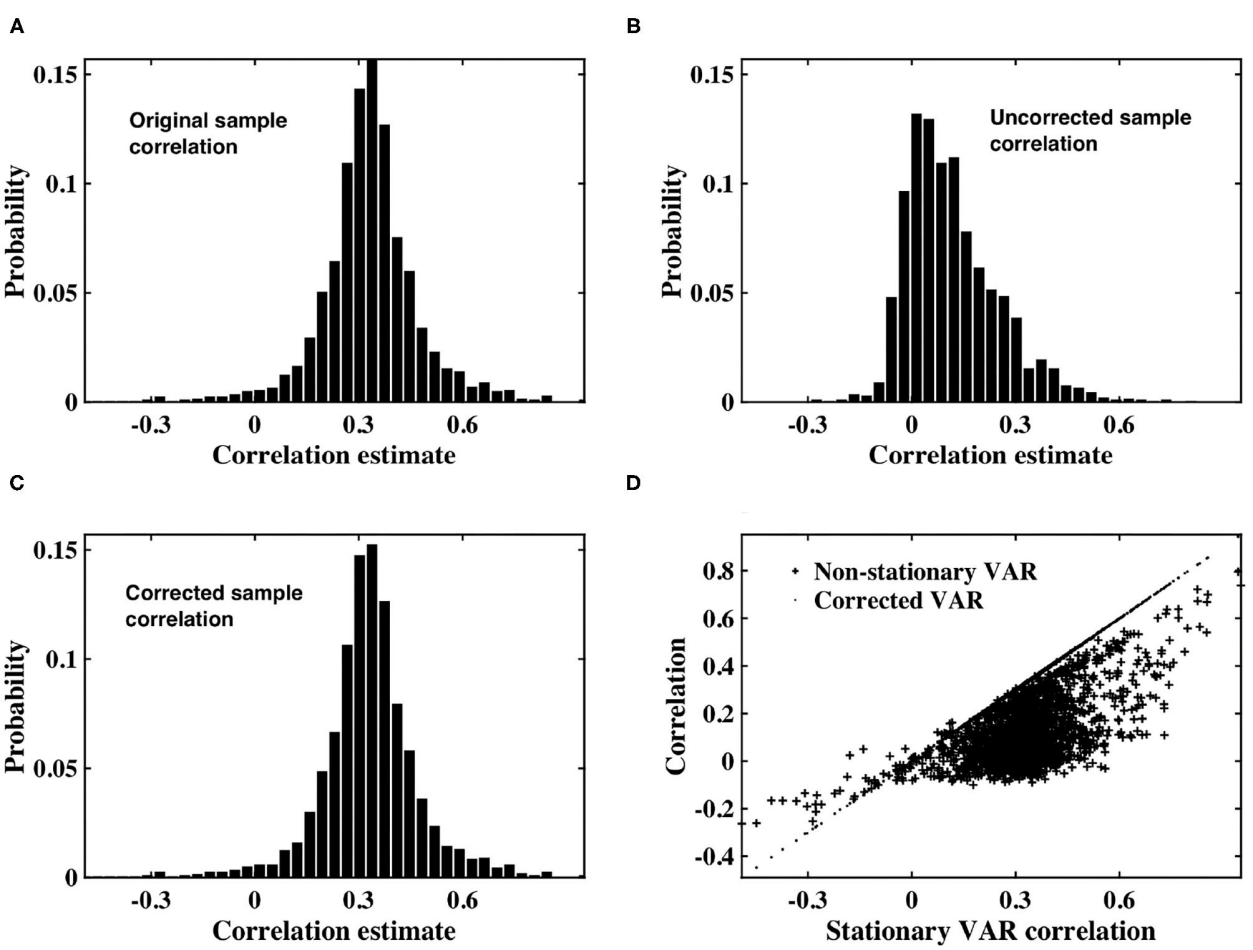
**(A)** Histogram of correlation estimates between stationary VAR timeseries pairs with underlying noise correlation, ρ = 0.3, according to Equation (1). **(B)** Histogram of correlation estimates acquired from non-stationary VAR timeseries pairs, with time-varying signal power for each timeseries within a pair randomly derived from *IG*(2, 5) or *IG*(3, 7). **(C)** Histogram of correlation between VAR timeseries pairs corrected for non-stationarity. **(D)** Scatter plots between original correlation estimates drawn from stationary VAR timeseries against correlation estimates of weighted, non-stationary VAR timeseries (crosses) and correlation estimates derived from timeseries corrected to restore stationarity (dots).

As shown in Figure 2, the distribution of sample correlation for the stationary VAR timeseries is larger than that of the white timeseries (Figure 1A), as expected due to the impact of the autocorrelation. The coefficients determining autocorrelation in the VAR timeseries were randomly generated, and hence had a variable impact on instantaneous correlation. Sample correlation between the non-stationary VAR timeseries (Figure 2B) is shown to be significantly impacted by the weighting of time-varying signal power. The mean correlation is reduced, showing that the slice dependent non-stationary variance has a destructive effect on correlation. After applying the precision correction the distribution of sample correlation is restored to that of the original stationary VAR timeseries (Figure 2C).

Figure 2D depicts a scatterplot of the correlation between the underlying stationary timeseries, and that estimated between the non-stationary VAR timeseries pairs, and the VAR timeseries pairs corrected using our proposed precision correction. The scatterplot shows that correlation between the non-stationary VAR timeseries almost always has magnitude less than that from which the stationary timeseries were generated. There are also many cases in which the sign of correlation between the non-stationary VAR timeseries has changed, from positive to negative or vice-versa. Importantly, sample correlation between the corrected timeseries has recovered to agree with the underlying correlation between the stationary VAR timeseries.

### 3.3. Experimental Results

#### 3.3.1. Non-stationarity of Resting State Data

**Figure 3:**
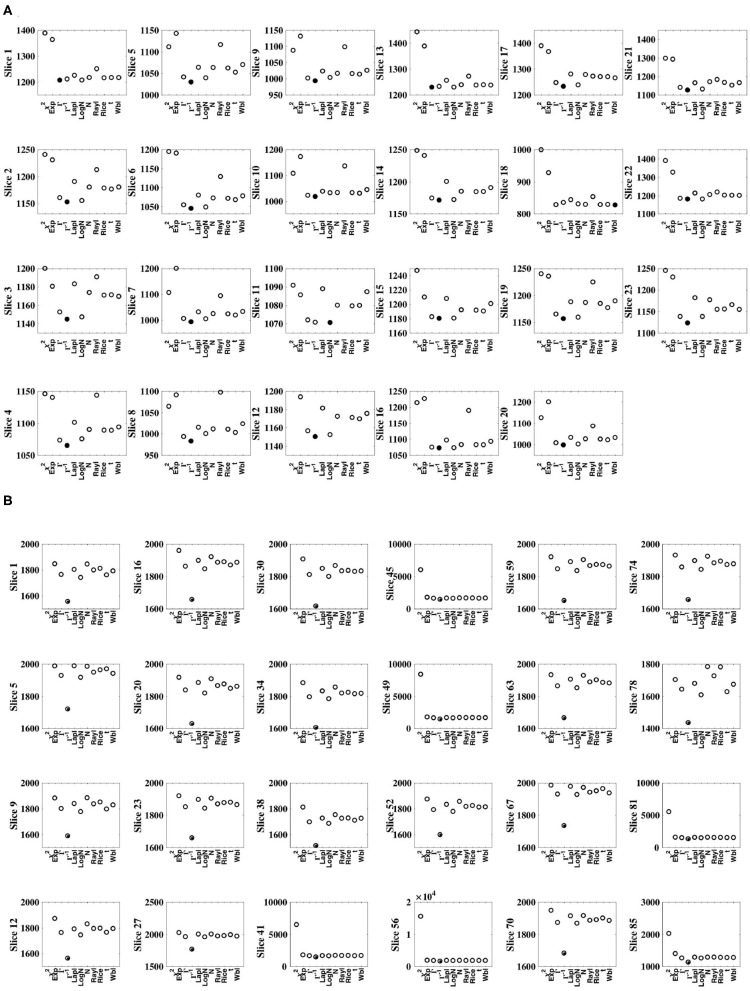
Comparison of MLE distribution fits to sample slice variance. Goodness of fit was evaluated using negative log likehood. The optimal fit is identified by a solid circle. **(A)** Shows results for dataset 1, while **(B)** shows results for dataset 2. In all cases, for both datasets, the inverse gamma distribution fits the data optimally.

**Figure 4:**
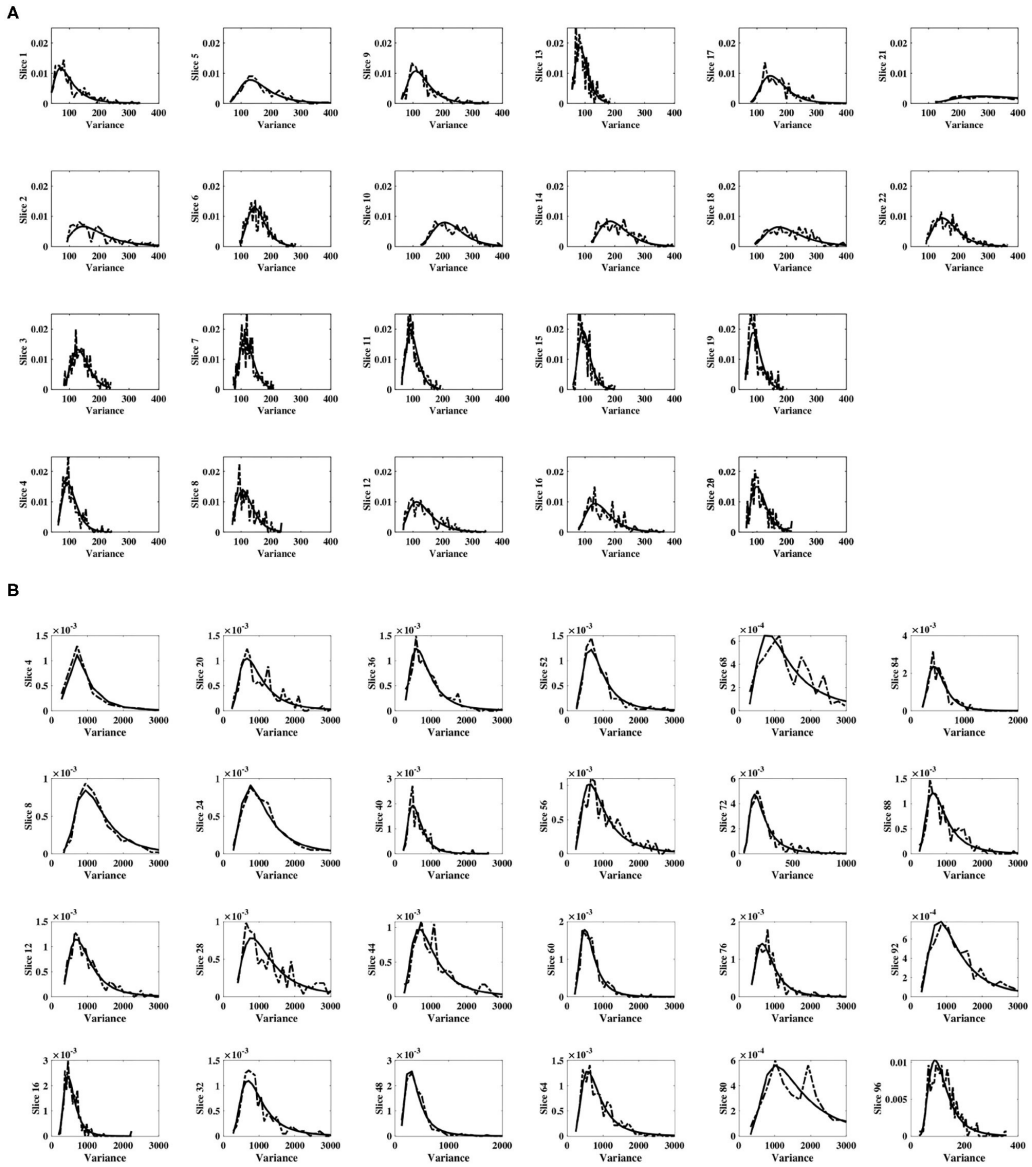
Distribution of sample slice variance of voxel intensities within each slice. Distribution of sample slice variance values within each slice (dashed) and MLE inverse gamma distribution fit to slice variance (solid), across slices. **(A)** Shows results for dataset 1, while **(B)** shows results for dataset 2.

For each subject, and for all slices within each subject's experimental dataset, the inverse gamma distribution best characterized the distribution of sample slice variance estimates of voxel intensities within the slice, as shown in Figure 3. For all slices, Figure 4 displays the distribution of sample slice variance, in addition to the MLE fit of the inverse gamma distribution to this experimental data. Despite significant variation in slice mean and variance, in each case the distribution of the sample slice variance is accurately characterized by an inverse gamma distribution.

**Figure 5:**
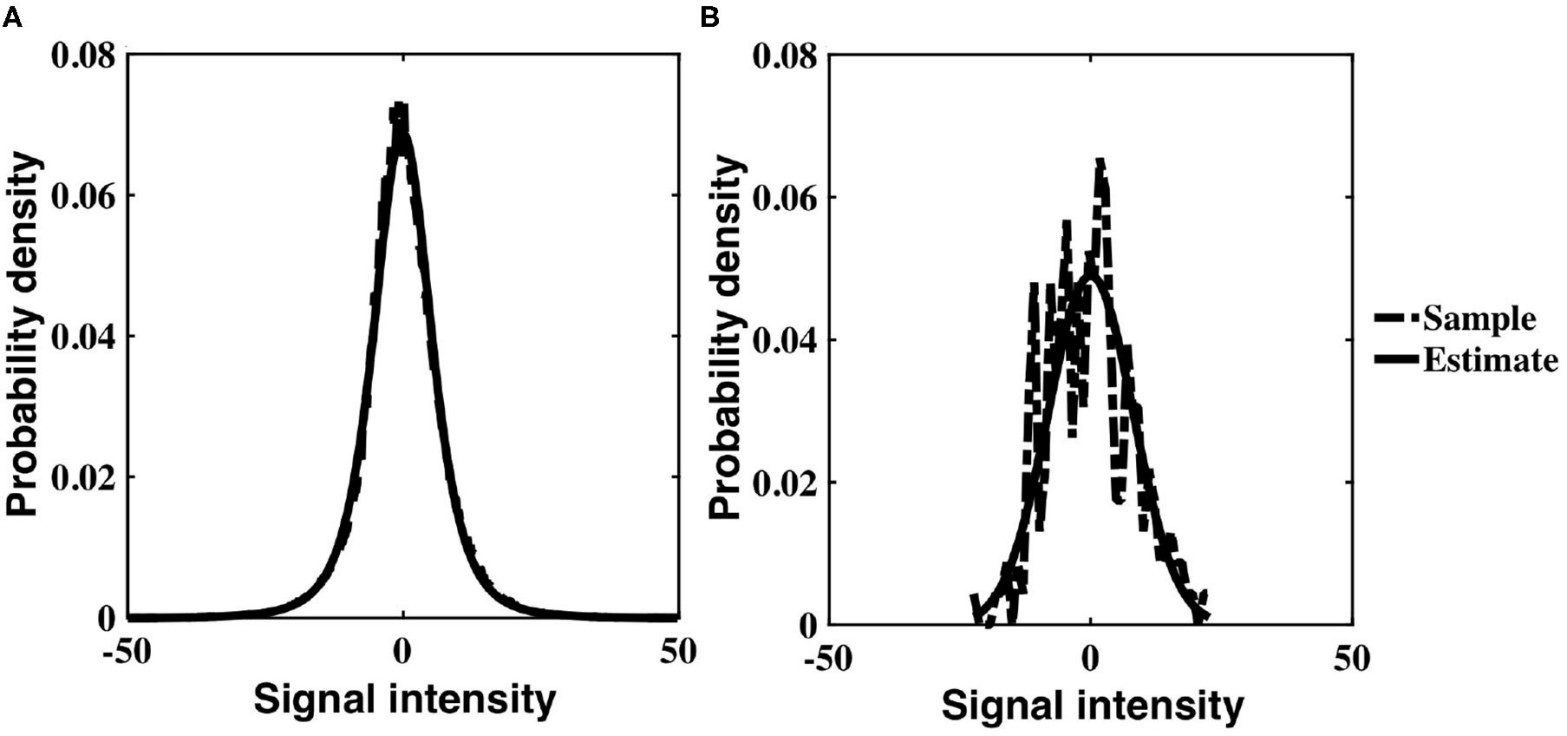
Three parameter Student's-*t* distribution (solid) fit to experimental data values (dashed) for a single subject, drawn from **(A)** all voxels within a slice, z = 16, and across time, **(B)** a single voxel timeseries within a slice (z = 16, x = 32, y = 32).

The probability distribution of Gaussian intensity values with an inverse gamma, non-stationary variance was analytically derived to be a generalization of a Student's-*t* distribution (Equation 6). Experimental intensity values corroborate this theoretical result; for each subject, the temporal distribution of voxel intensity values within a slice, as well as the distribution of individual voxel intensities, were optimally characterized by a three parameter Student's-*t* distribution. The resulting distributions for the slice and voxel are similar, although the voxel distribution has significantly reduced degrees of freedom (Figure 5). Since a Student's-*t* distribution is asymptotically Gaussian with degrees of freedom, our empirical results support the Gaussianity assumption inherent in a linear Gaussian model (LGM).

**Figure 6:**
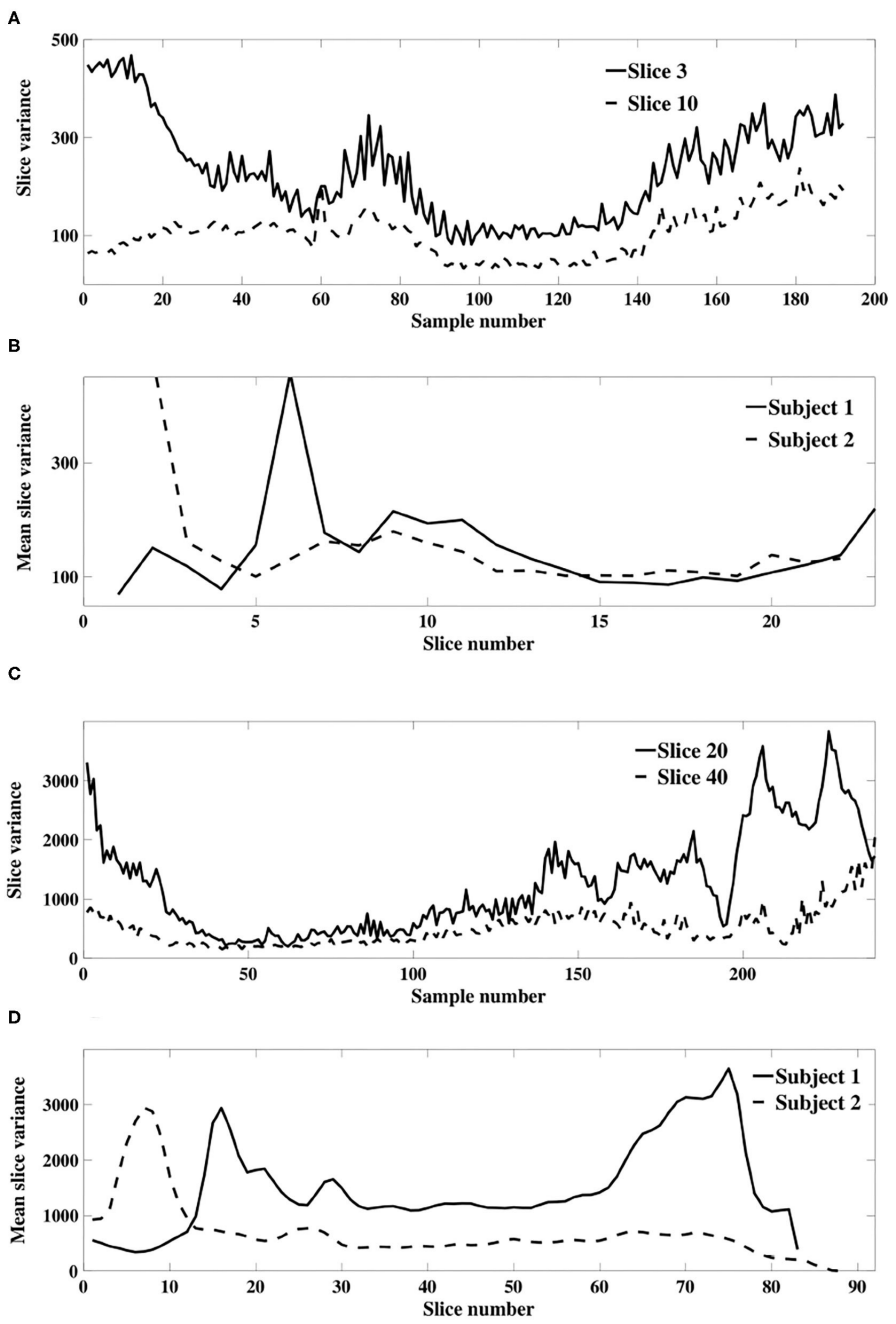
**(A)** Example sample slice variance from each resting state dataset. **(A)** (Dataset 1) and **(C)** (dataset 2) show calculated slice variance for exemplar slices from subject 1 of each dataset. **(B)** (Dataset 1) and **(D)** (dataset 2) show the mean sample slice variance across all slices of subject 1 and 2 in each dataset.

Our theoretical results in section 3.1.1 were derived using a model of slice dependent, time-varying variance. This model differs to that of Diedrichsen and Shadmehr (2005), in which a global time-varying variance was assumed so that all voxels within a 3D image were associated with a single non-stationary weighting. We verify the applicability of our model of slice dependent, non-stationary signal power using an experimental fMRI dataset. Figures 6A,C depicts the sample slice variance for two exemplar slices, demonstrating both the non-stationarity and the dissimilarity of the time-varying processes, and Figures 6B,D displays the mean slice variance for each slice across two different subjects.

**Figure 7:**
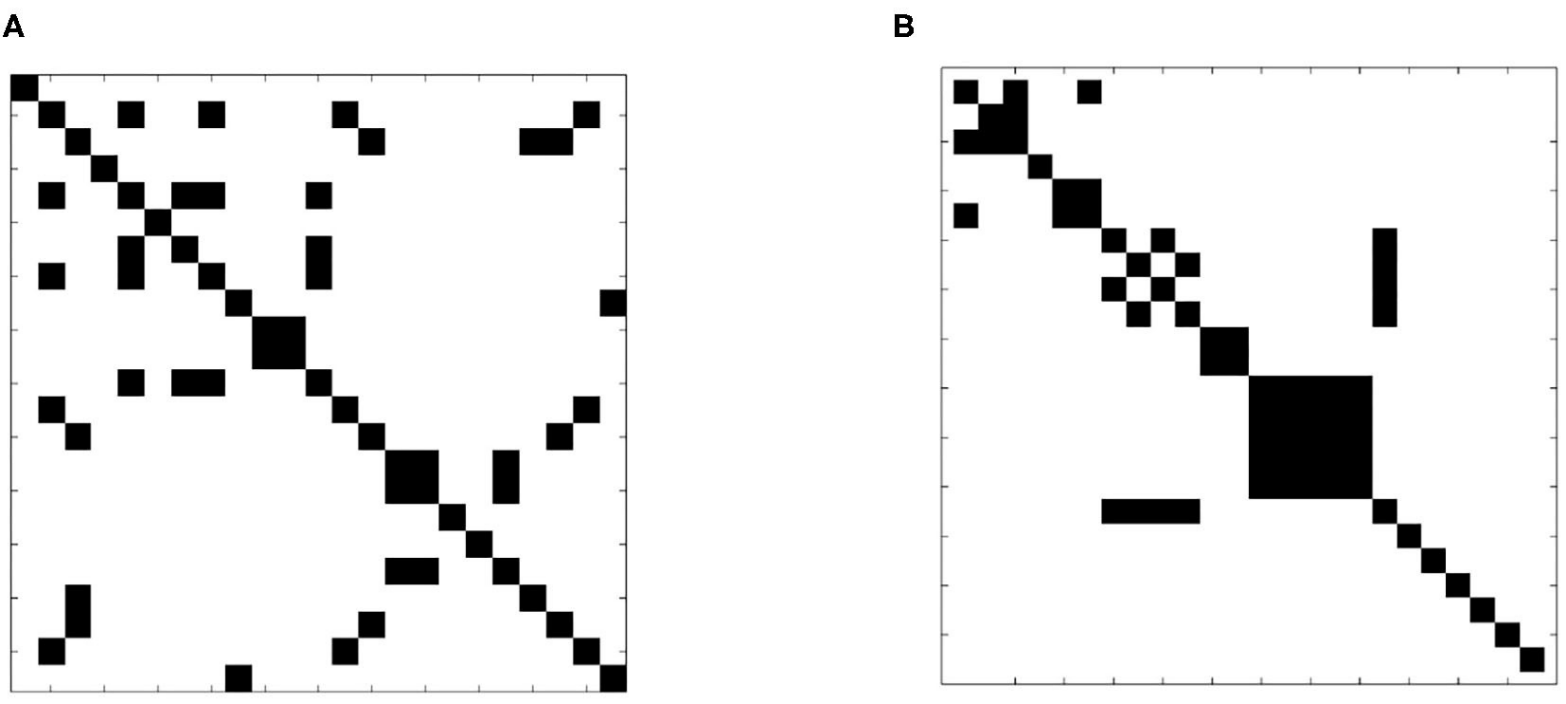
Test of distribution dependence between sample slice variance processes. The Wilcoxon signed-rank test was applied to each slice pair for each subject to identify dependence between sample slice variance distributions. **(A)** (Subject 1 from dataset 1) and **(B)** (subject 1 from dataset 2) show the results of each pairwise assessment. Black indicates that the null hypothesis of equivalence was supported whilst white indicates rejection of the null hypothesis (α = 0.01).

A more formal examination of dissimilarity was performed using the Wilcoxin rank-sum test to assess the equality of sample slice variance distributions. The test was applied to each slice pair; Figure 7 shows the result in matrix form. Black indicates that the null hypothesis of identical means was supported while white designates a rejection of the null hypothesis, indicating that sample slice variances for the pair were drawn from different distributions. The result clearly shows that slice variance distributions are dissimilar in most cases (93%), supporting our model of slice dependent non-stationary variance.

To determine if the slice dependence of the non-stationary weights was induced by differing ratios of tissue types in each slice, the linear association between slice time-varying weights, and each sample tissue variance timeseries, was examined. For all subjects, <10% of slices showed significant correlation with the sample tissue variance timeseries, and none of the inverse gamma parameters were significantly associated with the proportion of tissue type within each slice.

Stationarity of slices was evaluated by applying the augmented Dickey-Fuller (ADF) stationarity test to each slice. For all subjects, more than 70% of the sample slice variance timeseries were found to be non-stationary. None of the inverse gamma parameters were significantly associated with the proportion of tissue type within each slice.

#### 3.3.2. Impact on Connectivity

Non-stationary slice variance is problematic when methods that assume stationarity are employed to estimate brain connectivity. The theoretical results detailed in section 3.1.1 model non-stationarity derived from temporal changes in signal power. The impact of non-stationarity on correlation was analytically established in section 3.1.2, in which it was shown that a non-stationary weighting alters the expected value of sample correlation, which is a critical result for fMRI connectivity research. In this section, the theoretical results are corroborated with empirical evidence from both simulated and experimental data.

**Figure 8:**
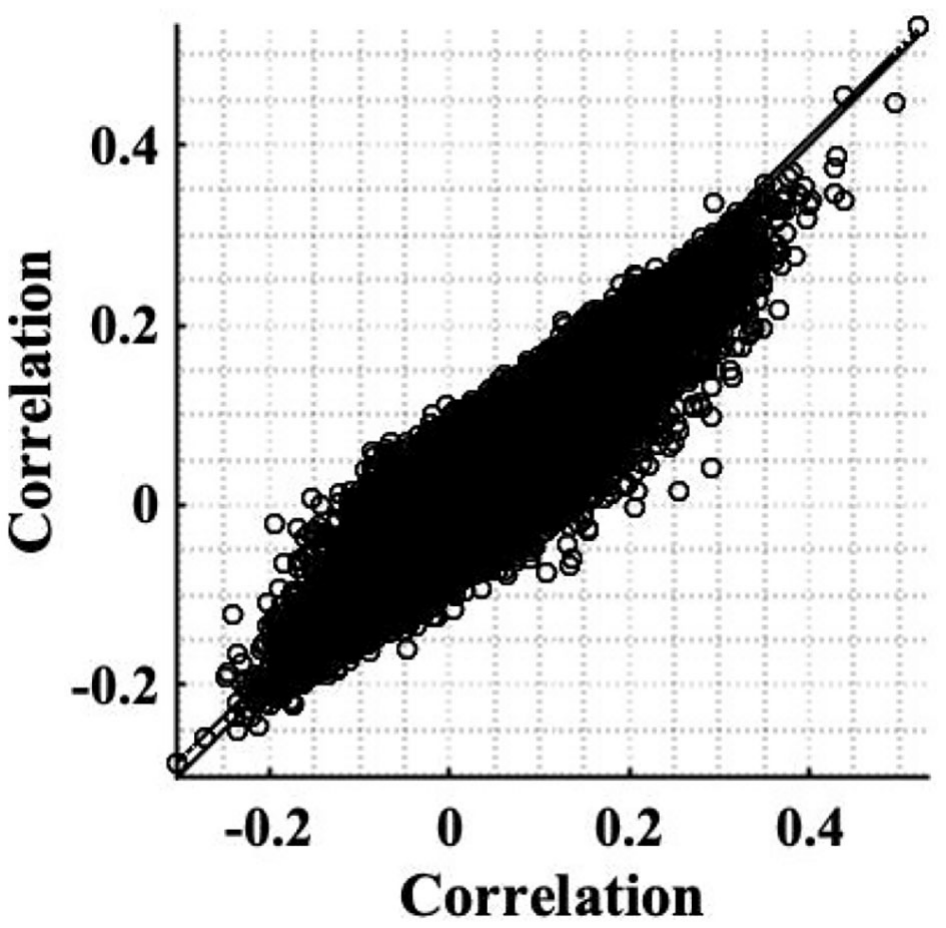
Scatterplot of correlation estimates between the RMC and brain voxels (slice 19), before correcting for non-stationarity, against correlation estimates acquired after applying the correction to restore stationarity. A line of best fit of pre-correction correlation, to post-correction correlation, values is shown (dashed), as well as the identity line denoting no change, to aid comparison (solid).

The simple timeseries correction (Equation 12), was applied to the experimental resting state dataset introduced in section 2.2 to examine its ability to restore stationarity of fMRI data. The weighting of correlation anticipated by the theory as a consequence of the non-stationary signal power (Equation 8), was found to be in close agreement with those found experimentally. For example, for voxels co-located with the RMC, the weighting anticipated by the sample slice variance was 0.8 for subject 1, while experimentally the change was 0.81. Furthermore, the scatterplot between experimental correlation estimates before and after correction, Figure 8 is reminiscent of the scatterplot generated using simulated data, Figure 1D. Although the spread of correlation in Figure 8 is greater, reflecting a more diverse range in underlying true correlation values, the principal eigenvector in the transformation of correlation between uncorrected timeseries, to correlation between corrected timeseries, clearly shows a size dependent amplification of correlation. This is highlighted by inclusion of the identity line in Figure 8, which designates where the eigenvector would lie if there was no change in the expected value of sample correlation between corrected timeseries. Note that the gradient of the eigenvector depends on the time-varying weights.

**Figure 9:**
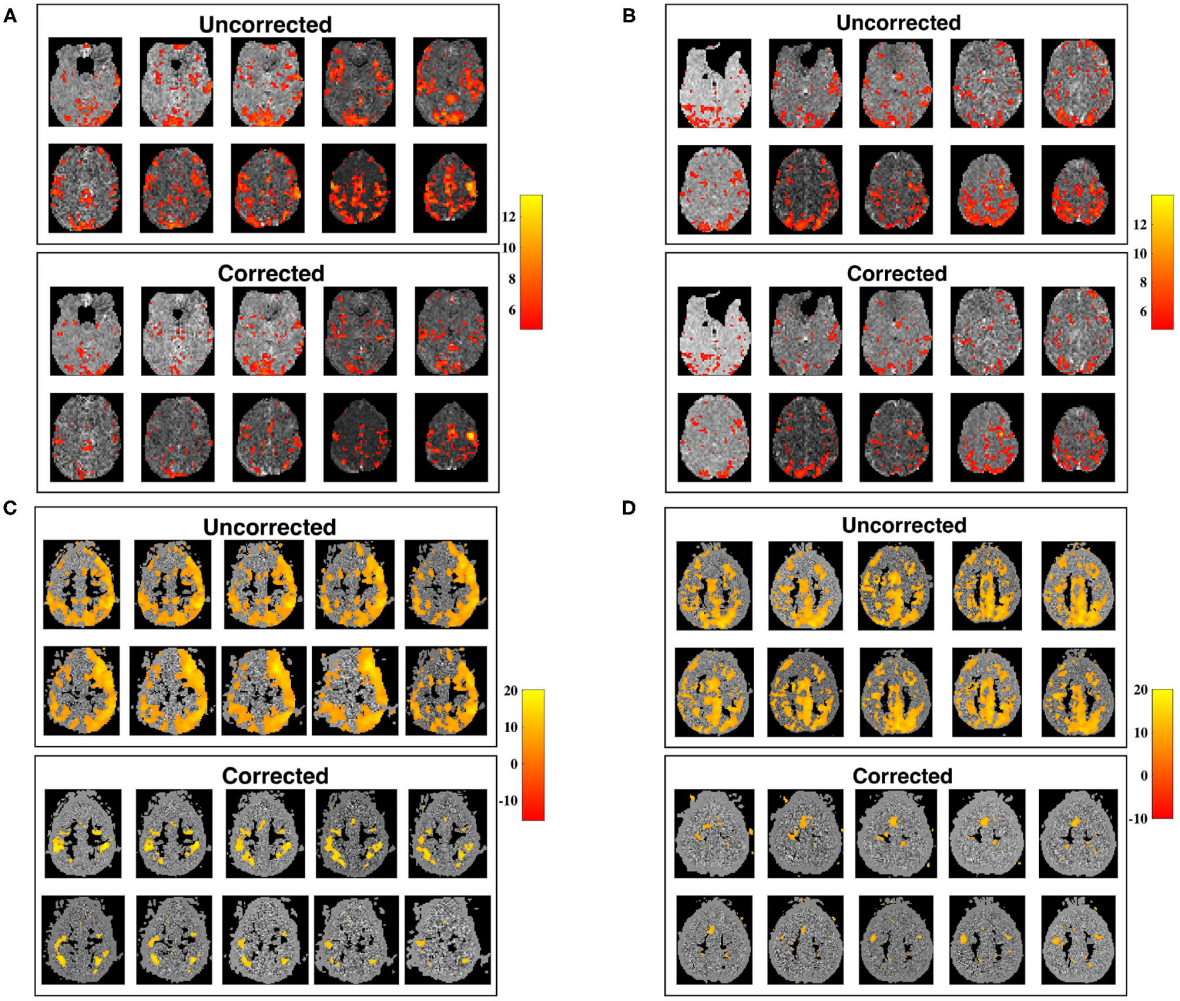
Slice maps of significant RMC-seed voxel correlation for uncorrected timeseries, and timeseries corrected for non-stationary signal power, tested using Fisher's z-transformation. **(A,B)** Show subjects 1 and 2 from dataset 1 while **(C,D)** show subjects 1 and 2 from dataset 2, before (above) and after (below) correcting for non-stationary signal power. For each subject, the number of outliers for corrected datasets is reduced. Spatial smoothing can cause clusters of outliers, which are visibly reduced after correction, creating significance maps in closer conformance with expected results.

The utility of our correction is demonstrated in Figure 9, in which seed-voxel correlation maps with a RMC seed are shown prior, and subsequent, to applying the correction for time-varying signal power (Equation 12), for two different subjects. The z-maps for both subjects show a visible difference in connectivity to the RMC as a result of applying the correction. Uncorrected z-maps contain a higher number of outliers than z-maps acquired using corrected timeseries, suggesting that uncorrected maps contain some connectivity artificially induced by the time-varying nature of voxel variance. This result agrees with the increased variance of sample correlation between non-stationary timeseries. If the significance tests of sample correlation are not altered to allow for this increased variance, spurious correlation will be erroneously identified as significant. Note that removal of non-stationarity changes both the mean and variance of sample correlation, as demonstrated in Figure 1, and thus the correction may appear as a change in threshold when applied to experimental data.

## 4. Discussion

Connectivity analyses of resting state fMRI data typically require stationarity of timeseries. However, this assumption is violated by non-stationarity derived from noise sources such as head movement, or by variable signal power originating from sources such as inhomogeneous RF amplification (Tanase et al., 2011) and signal attenuation (Stejskal, 1965). Violation of the stationarity assumption impacts connectivity results by implicitly applying a time-varying weight to timeseries elements. Random noise occurring at a sample associated with high variance may contribute more to a correlation estimate than a sample containing signal of interest but located in a region of low signal variance. Consequently, correcting for such time-varying signal power is not only desirable but necessary (Cohen et al., 2003).

We propose a model of non-stationarity motivated by time-varying signal power during acquisition. Since voxels within a slice are acquired simultaneously in 2D EPI acquisitions, we examined sample slice variance, describing temporal changes in the variance of voxel intensities within a slice. We established empirically that sample slice variance is non-stationary and can be well modeled by an inverse gamma distribution, in agreement with Hansen et al. (2003). Importantly, each sample slice variance process was found to be particular to that slice, contrary to the global assumption made by Diedrichsen and Shadmehr (2005). Incorrectly assuming an identical non-stationary process for all slices adversely affects connectivity results because signal of interest may be ignored based on an increase in noise variance in the overall image volume.

While signal non-stationarity can come from many potential sources, each impacting a different spatial extent, we chose to focus on slice specific variance due to striking observations of this in experimental results. Conversely, a voxelwise model of non-stationarity is difficult to substantiate and implement due to the need for sample variance to be calculated from multiple samples over space or time. Further, there is an infinite number of ways to separate the voxel's signal variance into spatial and temporal origins, that in the absence of prior or secondary information renders the approach intractable.

Our slice-specific method does not preclude the removal of regionally specific non-stationarity as a subsequent pre-processing step. The combination of the two would be confounded, as a given brain region may cross multiple slices, and would then contain voxels acquired with varying levels of signal power that should be removed first. The secondary analysis and removal of regional non-stationarity is beyond the scope of this paper, and research we intend to pursue in the future.

In establishing that the non-stationarity of voxel timeseries has a spatial dependence, it was shown that the time-varying weights for each slice are drawn from statistically significant distributions. We further established that there does not appear to be evidence for tissue specificity. Given that the slice dependence is dispersed across all tissue types, scaling timeseries by sample slice variance will not destroy dynamic connectivity. Consequently, additional methods can be employed to model time-varying connectivity.

Non-stationary signal power applied to voxel signals during acquisition has the propensity to change the distribution of the intensity values (Granger and Newbold, 1974). We have derived an analytic expression for the distribution of normally distributed voxel intensity, with non-stationary variance described by an inverse gamma distribution. The resulting distribution of voxel intensity was shown to be a generalization of a Student's-*t* distribution, which was further validated using an experimental resting state dataset. This result extends support to the normality assumption typically imposed by linear fMRI connectivity measures.

Having modeled the non-stationary distribution of fMRI voxel intensities, we subsequently considered the impact of non-stationary slice variance in the context of connectivity analyses, obtaining an analytic expression for correlation between non-stationary signals. Sample correlation between non-stationary timeseries was shown to derive from correlation between the underlying stationary signals, scaled by a function of the time-varying processes. Importantly, the scale factor was shown to have an absolute value less than unity. Consequently, slice dependent non-stationary variance processes diminish the strength of linear association between voxels. Furthermore, in the case where time-varying weights are drawn from the set of real numbers, the non-stationary process has the propensity to change the sign of association so that a positive correlation can become negative and vice-versa.

We derived the variance of sample correlation estimates between non-stationary timeseries. We demonstrated that time-varying power increases the variance. The theoretical results were corroborated with empirical results, which demonstrated a discernible change in the variance of sample correlation between non-stationary timeseries. This is significant as, if not accounted for, it will result in an increase in the incidence of spurious correlation. This may explain the large amount of correlation found in some fMRI datasets before correction, and the reduced amount of activation following correcting for time-varying signal power.

While this paper considers non-stationary signal power in resting state data, the result can equally be applied to activation data. The correction procedure has the propensity to change the signal means, however relative differences between signal means both spatially, in different brain regions, and temporally, will be preserved, and the linear relationship between the signal and the task will be preserved as the signal mean is an offset factor. This is the subject of our current work, and our preliminary testing confirms this supposition.

To address the impact of non-stationarity signal power on fMRI connectivity, a simple precision correction was proposed, which was analytically demonstrated to restore signal stationarity. The correction generated stationary signals that differ in value from the underlying stationary signals by a constant scale factor, which is inconsequential for correlation since it is insensitive to scale. The efficacy of our correction was validated using simulation data; after applying our proposed correction the mean value of sample correlation coincided with the underlying correlation between the stationary signals. Experimental data showed reduced outlier connectivity and the number of significant connections reduced in accordance with the smaller variance of stationary signals. Furthermore, the change in mean correlation agreed with that anticipated by the analytically derived expression for correlation between non-stationary signals. Note that removal of time-varying signal power will increase estimates of underlying linear connectivity since the time-varying weights diminish sample correlation estimates between timeseries.

The scope of current work has been to identify slice dependent non-stationary variance and propose a statistical correction for its presence in fMRI connectivity analyses. The 2D EPI datasets examined were both acquired in the standard axial slice direction. In order to probe the physical origins of non-stationarity, it will be of interest in future work to alter the slice direction to coronal, sagittal, or oblique slices. With B0 inhomogeneities more concentrated at the base of the frontal lobe, this is expected to change the signal power distributions when distributed across slices rather than confined within certain slices. Investigation into slice ordering, contiguous vs interleaved, and a comparison with 3D acquisitions are all potential ways in which the origins of the non-stationarity can be scrutinized. Further, it is of interest to explore Simultaneous Multi-Slice fMRI approaches that are increasingly popular for the increased temporal resolution they offer. The concurrent acquisition of multiple slices and un-aliasing of the slices via the receive array coils will likely render this a challenging statistical inference problem, but as with the current work, this is vital for the statistical integrity of the resultant brain connectivity maps.

## 5. Conclusion

Linear fMRI connectivity methods typically require stationarity of timeseries. We have demonstrated that resting state fMRI data do not meet this requirement but rather have time-varying, slice-dependent, variance that is well-modeled by an inverse gamma distribution. The resulting voxel intensity distribution under this model is a generalization of a Student's-*t* distribution. The impact of non-stationary signal power on connectivity was established by analytically deriving an expression for correlation between timeseries with time-varying variance. Subsequently, a correction was proposed and validated using both simulated and experimental datasets.

## Data Availability Statement

Requests to access these datasets should be directed to cedavey@unimelb.edu.au.

## Ethics Statement

All human imaging was conducted with the approval of the University of Melbourne Human Research Ethics Committee, and volunteers gave an informed consent prior to the experiment. The patients/participants provided their written informed consent to participate in this study.

## Author Contributions

LJ made the initial proposal to model variance using a time-varying inverse Gamma distribution. LJ and DG provided supervision and guidance on maths and data processing techniques. CD did the maths derivations and primarily implemented the Matlab coding and fMRI data processing. All authors contributed to the article and approved the submitted version.

## Conflict of Interest

The authors declare that the research was conducted in the absence of any commercial or financial relationships that could be construed as a potential conflict of interest.

## Abbreviations

ACF: autocorrelation function
AIC: Akaike's information criterion
AR: autoregressive
ARX: autoregressive exogenous input
BIC: Bayesian information criterion
BOLD: blood oxygen level dependent
CDGC: conditional directed Granger causality
CGGC: conditional Geweke's Granger causality
CIGC: conditional instantaneous Granger causality
CSF: cerebrospinal fluid
DCM: dynamic causal modeling
DGC: directed Granger causality
EEG: electroencephalography
EPI: echo-planar imaging
FIR: finite impulse response
fMRI: Functional MRI
FDR: false discovery rate
FPE: final prediction error
FWER: family-wise error rate
GC: Granger causality
GGC: Geweke's Granger causality
GLM: general linear model
GOF: goodness of fit
HQC: Hannan and Quinn's information criterion
HRF: hemodynamic response function
iid: independent and identically distributed
ICA: independent component analysis
IGC: instantaneous Granger causality
IIR: infinite impulse response
LFP: local field potential
LGM: linear Gaussian model
LISREL: linear structural relations
LMC: left hemisphere primary motor cortex
LSE: least squares estimation
MATLAB: matrix laboratory
MC: multiple correlation
MDL: minimum description length
MEG: magnetoencephalography
MLE: maximum likelihood estimation
MRI: magnetic resonance imaging
OSC: order selection criteria
PACF: partial autocorrelation function
PC: partial correlation
PCA: principal component analysis
PET: positron emission tomography
pdf: probability density function
PDGC: partial directed Granger causality
PET: positron emission tomography
RF: radio-frequency
RMC: right hemisphere primary motor cortex
ROC: receiver operating characteristic
ROI: region of interest
RSS: residual sum of squares
SEM: structural equation modeling
sICA: spatial ICA
sMRI: structural MRI
SNR: signal to noise ratio
SVD: singular value decomposition
tICA: temporal ICA
TR: repetition time
tSNR: temporal SNR
VAR: vector autoregressive
WGN: white Gaussian noise.
