# Supplementary material for: Correcting for Non-stationarity in BOLD-fMRI Connectivity Analyses
Source: Front Neurosci. 2021 Feb 24;15:574979. doi: 10.3389/fnins.2021.574979 (PMC7943734; doi:10.3389/fnins.2021.574979)
Supplement: Supplementary file 1 [file Data_Sheet_1.pdf]

A Appendix

A.1 Derivation of the distribution of non-stationary voxel intensity

We wish to determine the intensity distribution for a voxel with non-stationary noise, as defined in Eq. (5). The non-stationary noise process is inverse gamma distributed, according to Eq. (2).

Let $\alpha = \alpha_m$ and $\beta = \beta_m$ for readability, and introduce a precision parameter, $\tau_t = \frac{1}{\sigma^2_{m,t}}$, observing that Eq. (3) requires τ_t to be Gamma distributed according to, $\tau_t \sim Ga(\alpha, \beta)$ (Hamada et al., 2008, p.392), so that

$$p_{Ga}(\tau_t\,;\alpha,\beta) = Ga^{\alpha-1} e^{-\beta Ga} \frac{\beta^\alpha}{\Gamma(\alpha)}, \tag{1}$$

where Γ is the gamma function.

Let $z_t = \frac{x_{m,t}}{\sigma_x}$. Then Eq. (5) becomes

$$z_t \sim \mathcal{N}\left(0, \frac{1}{\tau_t}\right), \tag{2}$$

with conditional probability density function,

$$p(z_t \mid \tau_t) = \sqrt{\frac{\tau_t}{2\pi}} e^{-\frac{\tau_t}{2}(z_t)^2}. \tag{3}$$

The unconditional distribution of z_t is determined by integration over τ_t,

$$\begin{aligned} p(z_t) &= \int_0^\infty p(z_t \mid \tau_t)\, p(\tau_t)\, d\tau_t \\ &= \int_0^\infty \frac{\tau_t^{\frac{1}{2}}}{\sqrt{2\pi}} e^{-\frac{\tau_t}{2} z_t^2} \frac{\beta^\alpha}{\Gamma(\alpha)} \tau_t^{\alpha-1} e^{-\beta\tau_t} d\tau_t \\ &= \int_0^\infty \tau_t^{\alpha-\frac{1}{2}} e^{-\tau_t\left(\beta+\frac{z_t^2}{2}\right)} \frac{\beta^\alpha}{\Gamma(\alpha)} \frac{1}{\sqrt{2\pi}} d\tau_t. \end{aligned} \tag{4}$$

Substituting $a = \alpha + \frac{1}{2}$ and $b = \beta + \frac{z_t^2}{2}$ gives

$$\begin{aligned} p(z_t) &= \frac{\beta^\alpha}{\Gamma(\alpha)} \frac{\Gamma(a)}{b^a} \frac{1}{\sqrt{2\pi}} \int_0^\infty \tau_t^{a-1} e^{-\tau_t b} \frac{b^a}{\Gamma(a)} d\tau_t \\ &= \frac{\beta^\alpha}{\Gamma(\alpha)} \frac{\Gamma(a)}{b^a} \frac{1}{\sqrt{2\pi}}. \end{aligned} \tag{5}$$

Therefore,

$$\begin{aligned} p(z_t) &= \frac{\beta^\alpha}{\Gamma(\alpha)} \frac{\Gamma\left(\alpha+\frac{1}{2}\right)}{\left(\beta+\frac{z_t^2}{2}\right)^{\alpha+\frac{1}{2}}} \frac{1}{\sqrt{2\pi}} \\ &= \frac{\Gamma\left(\alpha+\frac{1}{2}\right)}{\Gamma(\alpha)} \left(\frac{1}{2\pi\beta}\right)^{\frac{1}{2}} \left(\frac{\beta+\left(\frac{z_t}{\sqrt{2}}\right)^2}{\beta}\right)^{-\left(\alpha+\frac{1}{2}\right)}. \end{aligned} \tag{6}$$

Substituting $x_{m,t} = z_t \sigma_x$ back into Eq. (6), orchestrated via a change of variables (Papoulis & Pillai, 2002), requires

$$\begin{aligned} p\left(x_{m,t}\right) &= p\left(z_t\right)\left|\frac{dz_t}{dx_{m,t}}\right| \\ &= \frac{\Gamma\left(\alpha+\frac{1}{2}\right)}{\Gamma(\alpha)}\left(\frac{1}{2\pi\beta}\right)^{\frac{1}{2}}\left(\frac{\beta+\left(\frac{\frac{z_t}{\sigma_x}}{\sqrt{2}}\right)^2}{\beta}\right)^{-\left(\alpha+\frac{1}{2}\right)}\frac{1}{\sigma_x} \\ &= \frac{\Gamma\left(\alpha+\frac{1}{2}\right)}{\Gamma(\alpha)}\left(\frac{1}{2\pi\beta\sigma_x^2}\right)^{\frac{1}{2}}\left(1+\frac{{x_{m,t}}^2}{2\sigma_x^2\beta}\right)^{-\left(\alpha+\frac{1}{2}\right)}. \end{aligned} \tag{7}$$

The voxel intensity distribution, Eq. (6), is an instance of the generalised Student's-t distribution (Härdle & Simar, 2007, p.129) with parameterisation $\mu_t = 0$, $\nu_t = 2\alpha$ and $\sigma_t^2 = \frac{\beta}{\alpha}\sigma_x^2$, where subscript t denotes a Student's-t distribution parameter. Consequently, the voxel intensity of sampled non-stationary resting state BOLD data described by the proposed model in Eqs. (2)-(3) is characterised by a generalised Student's-t distribution.

A.2 Derivation of the distribution of correlation between non-stationary time series

Sample correlation requires an estimate of the variance of each voxel timeseries. From the model for non-stationarity, the variance of a voxel timeseries can be determined as

$$\begin{aligned} \sigma_{x_{m,t}}^2 &= E\left\{{x_{m,t}}^2\right\} - E^2\left\{x_{m,t}\right\} \\ &= E\left\{x_{m,t}^{(st)2}\sigma_{m,t}^2\right\} - E^2\left\{x_{m,t}^{(st)}\sigma_{m,t}\right\} \\ &= E\left\{x_{m,t}^{(st)2}\right\}E\left\{\sigma_{m,t}^2\right\} - E^2\left\{x_{m,t}^{(st)}\right\}E^2\left\{\sigma_{m,t}\right\} \\ &= \mu_{x_{m,t}^{(st)2}}\mu_{\sigma_{m,t}^2}, \end{aligned} \tag{8}$$

where the last line results from the zero mean of the stationary voxel timeseries, Eq. (4).

To obtain an expression for sample correlation between voxel timeseries, it is useful to consider covariance between voxel intensity values and time-varying weights, which can be expressed as

$$\begin{aligned} \operatorname{cov}\left(x_{m,t}^{(st)}, \sigma_{m,t}\right) &= \frac{1}{T}\sum_{t=1}^{T}\left(x_{m,t}^{(st)} - \mu_{x_{m,t}^{(st)}}\right)\left(\sigma_{m,t} - \mu_{\sigma_{m,t}}\right) \\ &= \frac{1}{T}\sum_{t=1}^{T}x_{m,t}^{(st)}\sigma_{m,t} - \mu_{x_{m,t}^{(st)}}\mu_{\sigma_{m,t}} \\ &= \frac{1}{T}\sum_{t=1}^{T}x_{m,t}^{(st)}\sigma_{m,t} - \frac{1}{T}\sum_{t=1}^{T}x_{m,t}^{(st)}\frac{1}{T}\sum_{t=1}^{T}\sigma_{m,t}. \end{aligned} \tag{9}$$

Independence of voxel intensity values and time-varying weights requires covariance to be zero, so that Eq. (9) implies that

$$\sum_{t=1}^{T}x_{m,t}^{(st)}\sigma_{m,t} = \frac{1}{T}\sum_{t=1}^{T}x_{m,t}^{(st)}\sum_{t=1}^{T}\sigma_{m,t}. \tag{10}$$

To derive an expression for correlation between non-stationary voxel timeseries of the form Eq. (2), let y denote a non-stationary timeseries in slice n. Let $\operatorname{corr}\left(x_m^{(st)}, y_n^{(st)}\right)$ represent sample correlation between stationary voxel signals $x_{m,t}$ and $y_{n,t}$, and let $\operatorname{corr}\left(x_m, y_n\right)$ denote sample correlation between the weighted counterparts. Employing the definition of time-varying signal intensity in Eq. (2) and the expression for signal variance established in Eq. (8), sample correlation between

timeseries with non-stationary signal power can be expressed as

$$\operatorname{corr}\left(x_{m,t},y_{n,t}\right) = \frac{1}{T}\sum_{t=1}^{T}\left(\frac{x_{m,t}-\mu_{x_{m,t}}}{\sigma_{x_{m,t}}}\right)\left(\frac{y_{n,t}-\mu_{y_{n,t}}}{\sigma_{y_{n,t}}}\right)$$
$$= \frac{1}{T}\sum_{t=1}^{T}\left(\frac{x_{m,t}^{(st)}\sigma_{m,t}-\mu_{x_{m,t}^{(st)}}\mu_{\sigma_{m,t}}}{\sqrt{\mu_{x_{m,t}^{(st)2}}\mu_{\sigma_{m,t}^2}}}\right)\left(\frac{y_{n,t}^{(st)}\sigma_{n,t}-\mu_{y_{n,t}^{(st)}}\mu_{\sigma_{n,t}}}{\sqrt{\mu_{y_{n,t}^{(st)2}}\mu_{\sigma_{n,t}^2}}}\right). \tag{11}$$

Employing Eq. (10), and recognising that the stationary signals have zero mean, sample correlation between non-stationary signals becomes

$$\operatorname{corr}\left(x_{m,t},y_{n,t}\right) = \frac{1}{T}\sum_{t=1}^{T}\frac{\sigma_{m,t}x_{m,t}^{(st)}}{\sqrt{\mu_{\sigma_{m,t}^2}\mu_{x_{m,t}^{(st)2}}}}\frac{\sigma_{n,t}y_{n,t}^{(st)}}{\sqrt{\mu_{\sigma_{n,t}^2}\mu_{y_{n,t}^{(st)2}}}}$$
$$= \left(\frac{1}{T}\sum_{t=1}^{T}\frac{\sigma_{m,t}\sigma_{n,t}}{\sqrt{\mu_{\sigma_{m,t}^2}\mu_{\sigma_{n,t}^2}}}\right)\operatorname{corr}\left(x_{m,t}^{(st)},y_{n,t}^{(st)}\right)$$
$$= \frac{\mu_{\sigma_{m,t}\sigma_{n,t}}}{\sqrt{\mu_{\sigma_{m,t}^2}\mu_{\sigma_{n,t}^2}}}\operatorname{corr}\left(x_{m,t}^{(st)},y_{n,t}^{(st)}\right). \tag{12}$$

We now need to determine the variance of sample correlation between non-stationary time series. To do so, we first note that the sample variance of correlation between stationary time series is given by (Hooper, 1958)

$$\operatorname{var}\left(\operatorname{corr}\left(x_{m,t}^{(st)},y_{n,t}^{(st)}\right)\right) = \frac{1}{T}\sum_{t=1}^{T}\left(\frac{x_{m,t}^{(st)}y_{n,t}^{(st)}}{\sigma_{x_{m,t}^{(st)}}\sigma_{y_{n,t}^{(st)}}}-\frac{\mu_{x_{m,t}^{(st)}y_{n,t}^{(st)}}}{\sigma_{x_{m,t}^{(st)}}\sigma_{y_{n,t}^{(st)}}}\right)^2$$
$$= \frac{1}{T}\sum_{t=1}^{T}\frac{\left(x_{m,t}^{(st)}y_{n,t}^{(st)}\right)^2}{\sigma^2_{x_{m,t}^{(st)}}\sigma^2_{y_{n,t}^{(st)}}}-\operatorname{corr}\left(x_{m,t}^{(st)},y_{n,t}^{(st)}\right)^2$$
$$\approx \frac{1-\operatorname{corr}\left(x_{m,t}^{(st)},y_{n,t}^{(st)}\right)^2}{T-2}. \tag{13}$$

We now turn to the variance of sample correlation between non-stationary time series. Using the definition of sample variance gives

$$\operatorname{var}\left(\operatorname{corr}\left(x_{m,t},y_{n,t}\right)\right) = \frac{1}{T}\sum_{t=1}^{T}\left(\frac{x_{m,t}y_{n,t}}{\sigma_{x_{m,t}}\sigma_{y_{n,t}}}-\frac{\mu_{x_{m,t}y_{n,t}}}{\sigma_{x_{m,t}}\sigma_{y_{n,t}}}\right)^2$$
$$= \frac{1}{T}\sum_{t=1}^{T}\frac{\left(x_{m,t}y_{n,t}\right)^2}{\sigma^2_{x_{m,t}}\sigma^2_{y_{n,t}}}-\operatorname{corr}\left(x_{m,t},y_{n,t}\right)^2$$
$$= \frac{1}{T}\sum_{t=1}^{T}\frac{\left(\sigma_{m,t}x_{m,t}^{(st)}\sigma_{n,t}y_{n,t}^{(st)}\right)^2}{\sigma^2_{x_{m,t}}\sigma^2_{y_{n,t}}}-\kappa^2\operatorname{corr}\left(x_{m,t}^{(st)},y_{n,t}^{(st)}\right)^2$$
$$\approx \frac{1-\kappa^2\operatorname{corr}\left(x_{m,t}^{(st)},y_{n,t}^{(st)}\right)^2}{T-2}. \tag{14}$$

From Davey et al. (2013) the distribution of correlation will tend towards Gaussian, and consequently

$$\operatorname{corr}\left(x_{m,t},y_{n,t}\right) = \mathscr{N}\left(\kappa\operatorname{corr}\left(x_{m,t}^{(st)},y_{n,t}^{(st)}\right),\frac{1-\kappa^2\operatorname{corr}\left(x_{m,t}^{(st)},y_{n,t}^{(st)}\right)^2}{T-2}\right). \tag{15}$$

References

Davey, C. E., Grayden, D. B., Egan, G. F., & Johnston, L. A. (2013). Filtering induces correlation in fMRI resting state data. *NeuroImage*, *64*, 728-740.

Hamada, M., Wilson, A., Reese, C., & Martz, H. (2008). *Bayesian reliability*. Springer.

Härdle, W., & Simar, L. (2007). *Applied multivariate statistical analysis* (2nd ed.). Berlin: Springer.

Hooper, J. (1958). The sampling variance of correlation coefficients under assumptions of fixed and mixed variates. *Biometrika Trust*, *45*(3), 471-477.

Papoulis, A., & Pillai, S. (2002). *Probability, random variables and stochastic processes*. McGraw Hill.
